# Understanding the effects of education through the lens of biology

**DOI:** 10.1038/s41539-018-0032-y

**Published:** 2018-10-01

**Authors:** H. Moriah Sokolowski, Daniel Ansari

**Affiliations:** University of Western Ontario, London, ON Canada

## Abstract

Early educational interventions aim to close gaps in achievement levels between children. However, early interventions do not eliminate individual differences in populations and the effects of early interventions often fade-out over time, despite changes of the mean of the population immediately following the intervention. Here, we discuss biological factors that help to better understand why early educational interventions do not eliminate achievement gaps. Children experience and respond to educational interventions differently. These stable individual differences are a consequence of biological mechanisms that support the interplay between genetic predispositions and the embedding of experience into our biology. Accordingly, we argue that it is not plausible to conceptualize the goals of educational interventions as both a shifting of the mean and a narrowing of the distribution of a particular measure of educational attainment assumed to be of utmost importance (such as a standardized test score). Instead of aiming to equalize the performance of students, the key goal of educational interventions should be to maximize potential at the individual level and consider a kaleidoscope of educational outcomes across which individuals vary. Additionally, in place of employing short-term interventions in the hope of achieving long-term gains, educational interventions need to be sustained throughout development and their long-term, rather than short-term, efficacy be evaluated. In summary, this paper highlights how biological research is valuable for driving a re-evaluation of how educational success across development can be conceptualized and thus what policy implications may be drawn.

## Introduction

The education of children throughout their development is a key cornerstone for the creation of a successful society.^1–3^ In order to inform educational policymakers on how to maximize the success of educational strategies, various components of education systems have been extensively studied from a variety of perspectives, such as cognitive psychology, neuroscience, and genetics.^4–9^ Yet the causes that can help to explain why some children thrive while others fall behind in school remain unclear. A key factor in explaining why some children perform better than others in school is that children develop in heterogeneous environments and experience strikingly different education systems.^10–12^ Within the developing world, over 200 million children below the age of five experience poverty, with limited or no healthcare, poor nutrition, and inadequate education.^13^ These conditions of economic scarcity have been linked to negative outcomes across development, including decreased success in school.^14^ Consequently, researchers continue to advocate for policy changes to improve equity within and across education systems.^15^

The term equity has been confused with the notion of equality within the context of education. Researchers, educators, and policymakers have discussed this distinction in depth.^16–18^ Briefly, equality in education refers to the provision of equal resources and learning opportunities to all students. Although at a glance this seems fair, it has often been highlighted that some students need more resources than others to accomplish the same achievements. Therefore, equality of resources may not be fair, given that students enter with inequalities in capabilities and opportunities. Consequently, the concept of equity is more relevant. Equity in education is the notion of redistributing resources with the goal of eliminating systematic inequality of outcome measures, for example, giving low-income students access to exceptional teachers and additional funding to provide high-quality education to this population and ideally narrow achievement gaps. Indeed, a primary goal of education is to increase equity and close the achievement gap.

It has repeatedly been advocated that educational interventions should begin in early childhood in order to improve academic achievement in the long term.^13,19,20^ Economic reports suggest that applying early interventions to disadvantaged families will provide the greatest long-term rate of economic return to society.^21^ In the 1960s, research teams implemented multiple early intervention programs.^20^ These interventions ranged from small-scale, subject-specific interventions^22–25^ to intensive, large-scale public programs.^26–29^ In general, meta-analyses of effect sizes of these interventions report substantive improvements on educational outcome measures.^30,31^

Notwithstanding, pertinent concerns about the effectiveness of early education interventions remain because group differences persist, even after interventions (e.g., between children from families with low and high socioeconomic status (SES)).^20,32,33^ More specifically, although early interventions have been linked to improvements in educational achievement in children from families with low SES,^26–28^ these improvements were not large enough to eliminate group differences (i.e., close the gap between students from families with high and low SES).^32,33^ Additionally, individual differences within both high and low SES groups persist even following early educational interventions.^34–37^ Taken together, there is evidence to suggest that early educational interventions are not sufficient to compensate for unfavorable learning conditions experienced by many children. Although it is possible that further refining of interventions will improve their effectiveness, differences in a child’s predispositions coupled with their early pre-natal and post-natal experiences, likely also affect a child’s responsivity to specific educational interventions. Consequently, it may be more beneficial to modify the expectations and aims of early educational interventions. So, what goals and expectations for the effects of early educational interventions might be more realistic? In what follows, we discuss what is known about the effects of educational interventions both on individuals as well as the populations of individuals undergoing educational interventions as a whole. We then discuss how the study of genetics may inform our understanding of the effects of education. We close by discussing the implications of such data for educational policy.

## The alignment and misalignment between the goals and the effects of education

**Fig. 1:**
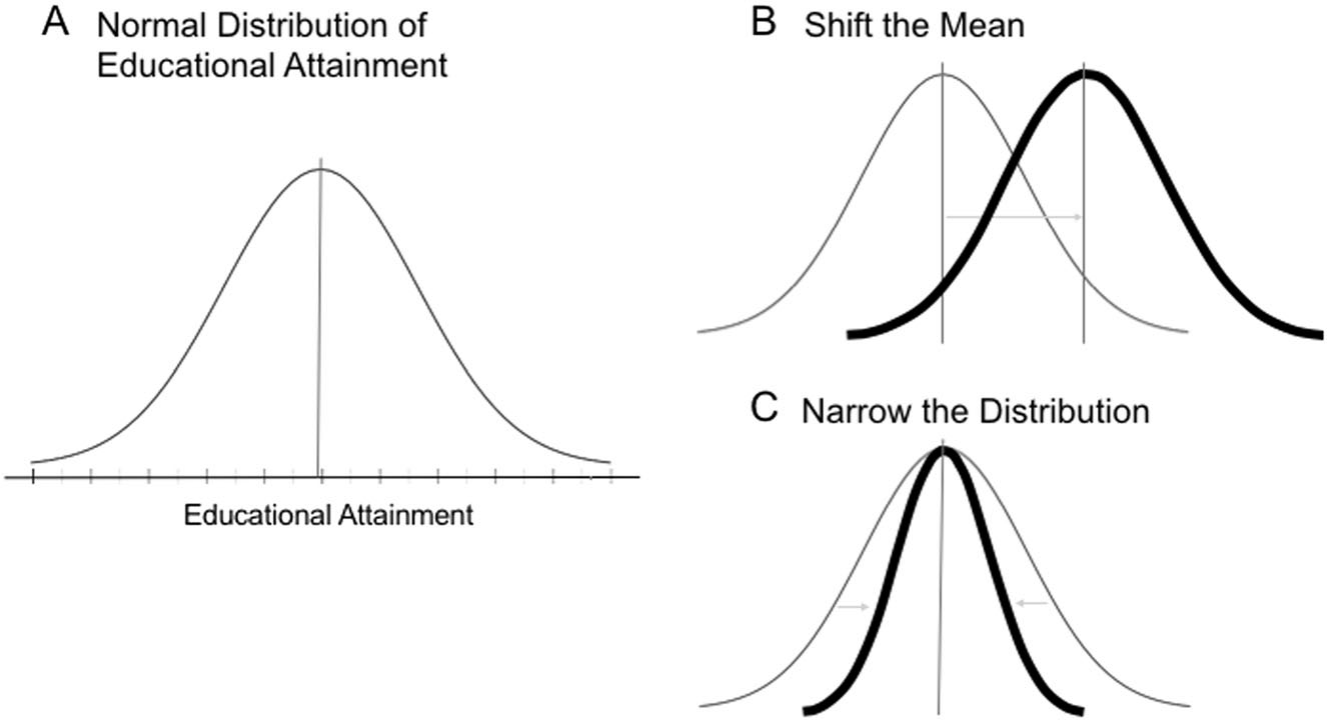
Aims of educational interventions are to shift the mean and narrow the gap of educational attainment outcomes. **a** Normal distribution of educational attainment. **b** Shift the mean. **c** Narrow the distribution to reduce the achievement gap between children on low and high ends of the distribution

Educational outcome measures, such as standardized tests of reading and mathematics, capture variability in performance that falls along a normal distribution in the population. This normal distribution describes how children within a population vary along an outcome of interest (Fig. 1a). This means that an individual child’s ability has a relative position compared to the other individuals in the population. The relative positions of an educational outcome measure for children in a population is referred to as the “rank order”.^36,38^ There are two main goals of early educational intervention programs: one goal is to help all children improve their scores (i.e., shift the mean of the distribution) (Fig. 1b). The other goal is to reduce the achievement gap between children on low and high ends of the distribution (i.e., narrow the distribution) (Fig. 1c). Though a laudable goal, early educational interventions may not actually narrow the distribution of educational attainment because they do not eliminate individual differences within populations,^34–37^ as pointed out by Scarr and McCartney^36^:

“One must distinguish environmental events that on the average enhance or delay development for all children from those that account for variation among children. There can be ‘main effect’ that account for variation among groups that are naturally or experimentally treated in different ways. Within the groups of children there still remain enormous individual differences, some of which arise in response to the treatment.^36^” Therefore, the existence of a rank order of abilities remains even after intervention (including when everybody has benefitted from the intervention).

Consider the follow-up study of children from low-income families who participated in the Carolina Abecedarian Project. This study revealed, even years later, that children who received a preschool educational intervention had higher academic achievement than children in an untreated control group.^39^ Indeed, the means for age-referenced standard scores from the Woodcock–Johnson psychoeducational battery reported in this study revealed that standard scores for reading, math, written language, and knowledge were greater in the experimental group than in the control group by 5–7 points. However, there was little difference in variance of scores in the intervention group compared to the control group. This shows that the gap between the highest and lowest scoring children was the same regardless of whether the group of children received the intervention.^39^ Although some children benefit greatly from interventions, other children do not show improvements. In other words, there are large inter-individual differences in response to interventions.

Importantly, this persistence of a rank order in the population does not mean that each individual child maintains their specific rank. Rather it means that individual differences persist. Indeed, children may respond differently to the interventions for a number of reasons. Figure 1 highlights one way that an intervention might narrow a distribution, namely, it is narrowed from both ends. However, it is certainly conceivable that interventions may change one end of the distribution. For example, in a basic reading intervention, a child who is already a proficient reader may benefit less than a child who has not yet learned how to read. This could depend on the match between the intervention and the individual’s ability. However, even children who begin an intervention with a similar skill level may respond very differently to the intervention. In empirical research on both reading and mathematics interventions, approximately 5% of the children were labeled as “treatment resisters”, because they did not respond to interventions.^34,35^

Critically, this means that a rank order will exist in the population before and after an intervention and there will be rank order of responsivity to an intervention. In other words, educational interventions do not eliminate individual differences in a population. As such, it is realistic to expect that a good educational intervention will shift the mean of a population but not drastically narrow the actual distribution of performance in a given educational domain. When thinking about the effects of education it is important not to confound inferences about individuals with inferences about a population of students within an educational system. The focus of this paper is on population-level effects of education and their constraints.

This distinction between the effect of an intervention on the mean compared to individual differences exists beyond the classroom or children in an education system of a particular country. International comparison studies show that there are mean differences in educational achievement between countries,^11,12^ but this does not imply that the shape of the distribution of performance on educational outcome measures is qualitatively different between countries. Indeed, at the population level, performance will be normally distributed in both countries, but the mean performance level may vary between countries. This has important implications for defining learning difficulties within and between countries. For example, if two nations with different mean achievement in mathematics both define children as having math learning difficulties when their math scores fall in the bottom 10% of the population, then the prevalence of math learning disabilities will be the same in both countries. However, the mean level of math achievement of children with so-called math learning difficulties in these two countries will differ. Thus, it is entirely possible for children who are considered to have math learning difficulties in one country to be within the normal range of performance given the distribution of another (on average more highly performing) country. Therefore, even though large differences in mean achievement between populations are possible, variability around the means likely remain unchanged.

## Biological mechanism underlying individual differences

What might explain this common finding that early educational interventions shift the mean of a distribution but do not eliminate individual differences in the population? The importance of biology and specifically genetics to understanding human behavior has been brought up for decades. Indeed, multiple authors have alluded to the importance of taking biology seriously within theories of development.^40–43^ In line with this, here we present research from the field of genetics that has the potential to shed light on the underlying biological mechanisms that may help us understand the effects of early educational interventions.

One of the oldest points of contention in the history of psychology is the nature-nurture debate.^44–46^ The nature side of this debate maintains that variation in behavior arises from genes only (G). The nurture side of this debate argues that variation in behavior comes from experiences only (E). However, decades of research examining both genetics and early experience have determined that treating nature and nurture as a dichotomy is nonsensical and futile.^47–51^ Currently, the question has shifted from the dichotomy of nature vs. nurture to questions about how an understanding of biology can inform our comprehension of the effects of nurture, such as in educational interventions. This has been referred to as “the nature of nurture”.^52^

In the social sciences, including psychology and education, there has been a strong bias toward the “nurture” perspective and a hesitation to consider “nature” as a possible factor that can explain individual differences in responses to educational interventions.^53^ One example of this strong bias toward a “nurture” perspective is the widely popular idea that changing the way an individual views their own learning can change his or her scholastic ability. Specifically, it has been argued that some individuals have a “fixed mindset”, meaning that they believe that their abilities are static, whereas other individuals have a “growth mindset”, and consequently, believe that their abilities can improve over time. Research has highlighted that individuals with a growth mindset are more likely to seek out challenges and persevere when faced with challenges.^54^ Converging psychological research has explored the personality trait called grit, defined by Duckworth as perseverance and passion for long-term goals. Research has shown that grit is a predictor of school achievement and later life success.^55,56^ These findings have led researchers and educators to try to improve academic achievement by fostering a growth mindset or encouraging grit. However, a recent meta-analysis examining the effectiveness of mindset interventions on academic achievement revealed that mindset interventions have only a weak effect on later academic achievement.^57,58^ One explanation for these weak effects is that the notion that individual differences can be overcome with quick fixes, such as thinking differently or approaching educational challenges with more grit, is problematic as it relies on misconceptions associated with the “nurture assumption”.^59–61^ This nurture assumption assumes that individual differences have no biological basis, and therefore can be easily overcome with interventions. However, the idea that a program can overcome individual differences contradicts what we know about the biological underpinnings of learning (see below) as well as the data on the changes induced by educational intervention (discussed above). These issues have been further highlighted in a recent blog post that discusses that “we should not forget that learners are all different and will have different strengths and weaknesses. Having a growth mindset does not mean that any weakness can be overcome”.^62^

This bias toward “nurture” and a disdain for explanations of individual differences that make references to biological factors may be explained by a misconception that the consideration of biological factors implies that behavior and individual differences therein are both determined and fixed. In what follows below, we analyze some of the key biological concepts that have been used to explain individual differences and try to dispel some of the common misconceptions associated with them.

### Heritability

One common misconception associated with research in genetics is that the term “heritability” means “genetic inheritance”. Heritability refers to how much variation in a trait (also called a phenotype) within a population arises from genetic variation among individuals. Heritability tells us nothing about what proportion of an individual’s phenotype is influenced by his or her genes. Moreover, measures of heritability can change even when the amount of genetic variation within a population remains constant. For example, research has revealed that SES modifies heritability of IQ in young children.^63^ Specifically, heritability of IQ increased as environmental variability decreased, and likewise heritability of IQ decreased when environmental variability increased. This study provides empirical evidence for the fact that heritability is simply the proportion of variance of a specific trait that is not explained by variance of the environment or random chance.

Estimates of heritability are most commonly derived from the study of twins. Specifically, by comparing the correlations in performance of genetically identical, monozygotic twins with that of non-identical, dizygotic twins, it is possible to indirectly estimate how much of the variability between individuals is influenced by genetic similarity. In such research, heritably is quantified as twice the difference in the correlations between monozygotic and dizygotic twins.^64^ While such studies provide a statistical estimate of the contribution of genetic variability to observed behavioral variability, they do not actually involve the measurement of the influence of genes. Heritability is a population measure, not a causal process within a single individual.

Another misconception is that heritability is negatively related to plasticity or modifiability. High heritability measures do not indicate that the behavioral trait within individuals is fixed and cannot be changed by the environment or that a certain behavioral trait influenced by a particular genetic variant is determined. Importantly and perhaps counter-intuitively, a higher heritability estimate implies greater equity of an environment.^65^ If the heritability of a trait is 100%, this invariably means that there is no variation in the environment (i.e., the environment is equitable). It is important to note here that this notion of environmental equity does not say anything about the quality of the environment. Indeed, if a poor education system is implemented in exactly the same way across a population it is equitable and the heritability of educational achievement would be close to 100%.

It is especially important within the context of education to be cognizant of the fact that research indicating that educational achievement has high heritability does not mean that achievement is determined. Indeed, effects of heritability are probabilistic, not deterministic. Meta-analytic evidence has revealed that genetic effects vary across contexts.^66^ Specifically, Tucker-Drob and Bates’ meta-analysis revealed that in the United States, where educational achievement is strongly correlated with SES,^67^ heritability varies as a function of SES. In contrast, countries with social policies that ensure more uniform access to high-quality education (such as countries in Western Europe and Australia), exhibited no interaction between heritability and SES. In other words, educational achievement is reported to have greater heritability in populations from countries with greater equity in educational policy.^65,68^

### Genetics and educational achievement

The commonly replicated finding from twin studies that educational attainment is heritable has driven researchers to examine the associations between the human genome and educational outcome measures.^9,69–74^ Recently, it has become possible to measure variability in genes across the entire genome of individuals and then relate this variability in the actual genes to behavioral variability.^75–78^ Genome-Wide Association Studies (GWAS) use a complex statistical method to identify a set of genetic variants from across the entire human genome (all of our DNA) that correlate with a behavioral outcome measure such as educational attainment (for a detailed description of GWAS, see ref. ^79^). Typically, each GWAS examines millions of genetic variants (also known as single-nucleotide polymorphisms (SNP)) simultaneously. Notably, these “millions” of SNPs are not independent as they are often linked with each other via linage disequilibrium.^80^ Researchers use this data to identify genetic variants that may contribute to individual differences in the population on a behavioral trait of interest. A major advantage of GWAS is that it is an unbiased approach that relies on genetic variants across the entire genome that correlate with the outcome measure, rather than a priori choosing particular genetic variants (i.e., the candidate gene approach^81^). A critical disadvantage of GWAS has been a lack of reproducibility. Specifically, it was found that the genetic variants associated with a trait in one study were not consistently related to that same trait in other studies.^82,83^ Reasons for this lack of reproducibility, such as insufficient sample sizes, lack of sufficient statistical tools to control for the extensive multiple comparisons, environmental differences between the cohorts, and lack of specificity in measuring behavioral outcome measures, are beyond the scope of this paper (for review, see refs. ^79,82–84^). Recently, progress has been made to overcome these limitations by increasing the sample size used to estimate the correlation between genetic variants and complex, behavioral phenotypes. Indeed, researchers have used GWAS to link genetic variants to individual differences in educational attainment measures such as number of years of schooling completed,^9,70^ intelligence (IQ),^69,71^ and even more specialized cognitive measures such as reading^74^ and math ability.^73^ The relation between “years of education” and the three SNPS from the original GWAS measuring “years of education”^70^ was replicated several years later in a study that increased the sample size threefold.^9^ Critically, the estimated effects sizes of these three SNPs are small (coefficient of determination R(2) ≈0.02%; this equals approximately 1 month of schooling per allele). Although, this research is in the early stages, GWAS research has revealed that contrary to previous theories based on candidate gene effects, many genes, each explaining a tiny proportion of variance, correlate with outcome measures.

More recently still, researchers have started to use a set of genetic variants (usually SNPs) selected from the entire genome using results from GWAS as predictive measures. Specifically, data from GWAS is used compute a composite genetic score for a set of genes. This is often referred to as a genome-wide polygenic score (GPS), and relates to a specific trait.^72^ A GPS is a number that is composed of a set of weighted genetic variants (i.e., weighted SNPs) across many genetic loci that best predict a specific trait, such as educational attainment. Using GWAS to delineate the polygenic predictors of an outcome measure, the GPS score can be used with a different, smaller sample of individuals, to predict more refined outcome measures. For example, this method has been used to link genetic variants from a large GWAS study to specific measures of educational attainment.^9,70,72^ Belsky et al.^72^ computed a GPS for each individual of the “Dunedin cohort” in New Zealand for educational attainment. This GPS was derived from the GWAS that identified molecular genetic predictors of “years of education” in more than 100,000 individuals^70^ and was replicated in a larger sample of almost 300,000 individuals.^9^ This GPS score for educational attainment was computed by summing all alleles that were associated with “years of education” across many genetic loci that are weighted by effect sizes that were estimated from the Okbay et al.^9^ GWAS study.^85,86^ This polygenic score predicted adult economic outcomes and behavior across the life span.^72^ In another study, a GPS derived from the Rietveld et al.^70^ explained up to 9% of variance in educational achievement scores at ages 7, 12, and 16.^87^ This GPS score was also associated with general cognitive ability and family SES. However, there was no evidence for GPS interacting with general cognitive ability or SES to predict educational achievement. Most recently, a GWAS study with 1.1 million individuals identified 1271 independent SNPs associated with educational attainment.^88^ These SNPS were used to compute a polygenic prediction score that explained 11–13% of the variance in educational attainment and 7–10% of the variance in cognitive performance in independent samples.^88^ Although the novel techniques used in these studies are still in their infancy, they provide the valuable insight that there is undoubtedly a relationship between individual differences in genetics and variability of educational achievement. Approaches like GPS help us to understand that biology plays a critical role in explaining individual differences in important life-span outcomes.

In the context of the present discussion, these data provide compelling evidence that the common assumption that nurture can eliminate individual differences in a population contradicts the biological mechanism associated with learning. Indeed, although the experience of educational interventions certainly affects educational outcome measures, individual differences in ability cannot be entirely attributed to the educational environment. This is due to the fact that biological factors play a key role in explaining individual differences in academic achievement. Therefore, the relation between genes and educational achievement further highlights the need to conceptualize the distinction between the two goals of educational interventions, namely, to shift the mean and narrow the distribution.

### Gene–environment interplay

While GWAS studies and resulting GPS scores are useful to better understand the contributions of biology to behavioral outcomes, it is important to reiterate that genes and the environment do not operate independently.^89^ Indeed, considerations of these genetic influences on individual differences in educational outcomes should still not be equated to assuming that these traits are inherited and therefore fixed.^49^ A trait being 100% heritable does not mean that that this trait is completely explained by differences in individual’s DNA sequence because genes do not operate independently from environmental influences. Instead, complex dynamic interplay between genetic predispositions and environmental exposure (gene–environment interplay) lead to different developmental outcome measures.^50,90^ For decades, scientists have explained that while genes (i.e., an individual’s DNA sequence) and the environment (i.e., experience across the life span) may seem like independent components that uniquely affect phenotypes, this is not the case. It is not either genetics or the environment that influences behavior, it is both. Moreover, the additive effect of genes plus environments (G+E) is not sufficient to explain individual differences in phenotypes. The presumed inverse relationship between genetics and environmental factors ignores large and distinct bodies of research that highlight that genes and experiences modify the effect of each other on traits.^51,79^ Therefore, the dynamic complex relationship between genetic and environmental factors leading to specific outcomes across development lead researchers to develop the general term “gene–environment interplay”.^50,79,91,92^

In what follows, we discuss how several components of “gene–environment interplay” relate to educational attainment. Gene–environment interplay is a broad term that incorporates the effect of genetics, gene–environment correlations, and gene–environment interactions. These biological mechanisms that underlie gene–environment interplay across developmental time will be used to help explain why individual differences remain even after early educational interventions.

### Gene–environment correlations and the fade-out of educational interventions

Scarr and McCartney proposed a theory of development that genotypes direct experience. More specifically, the authors suggest that an individual’s genetics predicts their behavior across development both directly and through experience.^36^ Although this important theoretical contribution is not without criticism and counterarguments,^93,94^ it sets a critical foundation for the way that the link between genes and environments relate to predict behavior.

Indeed, an individual’s genetics and environment correlate in different ways across development. Gene–environment correlations can be passive, evocative, or active. Passive gene–environment correlations result from parents creating an environment that is influenced by their own heritable traits. Notably, under these conditions, the effect of the child’s genotype is constrained. Evocative gene–environment correlations result when an individual’s heritable behavior evokes an environmental response. Active gene–environment correlations occur when an individual possesses a heritable inclination to select a specific environment. The relative importance of these gene–environment correlations is hypothesized to change across development.^36^ Passive gene–environment correlations may influence behavior more strongly in infancy and early childhood, whereas evocative and active gene–environment correlations may become more important during later childhood and adolescence. This is because, later in life, children can select niches that best fit their genotype. In contrast, a young infant is unable to select environments that fit their genotype and therefore passive gene–environment correlations dominate early in development. Consequently, in the capacity of gene–environment correlations, the environment may play a greater role in later childhood and adolescence compared to infancy and early childhood.

These developmental changes in the nature of gene–environment interplay are important to consider in the context of what is known about the long-term efficacy of early interventions. Research on the long-term effects of early education has revealed that, although interventions shift the mean of the normal distribution of an educational attainment outcome measure in the short term, the distribution eventually shifts back toward the pre-intervention mean (Fig. 1a). Consistent with this, it has been found that the long-term results of early interventions are disappointing because the striking short-term improvements in children’s school success fades over time,^20,95,96,97,98^ suggesting that investment in early educational intervention programs does not always lead to long-term improvements in later school and life success. Until recently, the mechanisms underlying fade-out effects were an enigma. However, recent work reveals that a large proportion of fade-out effects in a math intervention are explained by pre-existing differences (such as SES and academic ability before the intervention) between children, rather than school factors such as low levels of classroom instruction.^95,99^ These data suggest that the long-term math outcomes of children are strongly influenced by individual stable traits across development. These findings highlight the need to consider how biological predispositions correlate and interact with the dynamic educational environment. In particular, it is plausible that developmental changes in gene–environment correlations could, in part, explain the frequently observed fade-out of educational interventions. Specifically, young children who, as a population, have a comparatively limited ability to select their own environment (e.g., they have to attend school, their parents have substantial control over their behavior, etc.: passive genotype–environment correlation) are more strongly influenced by environmental inputs, even if these do not align with their genotypes. This is a potential explanation for the observed short-term gains and a shift in the overall mean of the distribution following an educational intervention. Over developmental time, however, children begin to select environments that more closely fit their genotypes and thus select environments that play to their intrinsic strengths and abilities, leading to a less pronounced effect of the environment and thus the maintenance of the effects of educational interventions. This shift from passive to active gene–environment correlations across developmental time may be important for understanding the regression of the mean back to pre-intervention levels. Together, research on gene–environment correlations is among the most compelling evidence for how examining the effects of educational interventions through the lens of biology is beneficial for understanding how children experience and respond to educational interventions.

Gene–environment correlations have been reported for phenotypes associated with educational outcomes. For example, the polygenic score for educational attainment, discussed above, correlated with later life success of the individual as well as the SES of the home into which the individual was born.^72,100^ This research supports the notion that children with certain genotypes may be more likely to receive certain kinds of parenting, evoke certain responses, and select certain aspects of environments.

In line with the notion of gene–environment correlations, a recent study has examined whether the parents’ genetic variants that are not passed on to the child affect the child’s educational attainment.^101^ Specifically, a GWAS study of educational attainment^9^ was used to compute polygenic scores for parents that only included genetic variants that were not passed on (i.e., non-transmitted genetic variants) to the children. The study revealed that the polygenic score that was computed using non-transmitted genetic variants accounted for approximately 30% of the variance in the children's educational achievement that was explained by the polygenic score calculated using the transmitted variants. The researchers concluded that genetic variants in parents that are not passed onto the child affect the educational attainment of the child. They call this indirect effect on educational attainment “genetic nurture”.^101^ Findings such as these illustrate that it is simply impossible to separate nurture from nature. In this case parental genes, which are not passed onto the offspring, shape the environment that the child experiences. This finding is very much in agreement with the notion put forward by Scarr and McCartney^36^ that “genes direct the course of human experience”.

There is no doubt that the use of polygenic scores in conjunction with environmental influences is useful for increasing understanding of the complex link between genetics and educational attainment. However, it has also been suggested that society can benefit from using information about a polygenic score in education. A recent paper indicated that polygenic scores “could be useful in both society and science to estimate genetic potential as well as risk in relation to all domains of functioning, including cognitive abilities and disabilities, personality and health and illness” (p. 1373).^102^ This could be useful specifically within the context of education for identifying biological mechanisms that might help educators to understand why an individual student is struggling in a certain educational domain. Importantly, considering polygenic scores may also help educators to think more carefully about the environment. For example, one child may have a high polygenic score but nevertheless struggle in school, whereas another child may have a low polygenic score but excel at school. These contrasting examples should motivate an analysis of the environments that these children are experiencing. Moreover, knowledge of the environments experienced by these children may increase our understanding of which environments buffer against the effects of genetics or prevent the expression of the biological constraints that a learner brings to an educational setting. Unsurprisingly, there are societal fears that stem from concern that genetic scores may be used for eugenics purposes.^103^ Eugenics is a term coined in the late 1800s that refers to the idea that genetics can be used to control breeding to increase the quality of a human population by increasing the occurrence of desirable heritable traits.^104^ These societal fears about using polygenic scores for improving education are based in the misunderstanding that theses score are deterministic rather than probabilistic. Indeed, a base knowledge of the interplay between genetics and experience is a necessary foundation to support the valuable discussion of the application of genetic sciences to tailoring interventions to individual learners.

### Gene–environment interactions

Critically, experiences, genetics, and the way that they correlate with one another is not the whole picture. Indeed, children arrive at school with more than just a set of genes and countless experiences that correlate. Specifically, in addition to gene–environment correlations, recent work has revealed complex dynamic interactions between genetic predispositions and early environments. These interactions highlight the non-deterministic way that genes affect behavioral outcomes across development.^72,73,75,78,105–107^ Everyone has DNA sequences that respond to countless experiences (i.e., environments) across the lifespan. A span of DNA that comprises a gene is responsive to the environment. Specifically, it produces more or less of its gene product (e.g., RNA, protein) depending on experience. Early adversity has been linked to negative biological and psychological outcomes across development.^108,109^ A growing body of research has indicated that genetic predispositions interact with environmental exposure to affect behavioral outcomes.^47,50,51^ This can be conceptualized as genetic predispositions moderating the relationship between early experience and later phenotypic outcomes. The gene–environment interaction field has come under criticism in regard to sample sizes used, varying assessments of environments and behavioral outcomes, and inconsistent statistical methods.^48^ Despite these important limitations, this body of research highlights that to understand individual differences in behavior, it is critical to consider the way that children’s genetic prepositions interact with their early experiences across developmental time. Therefore, it may be fruitful to explore the effect of gene–environment interactions on different components of educational attainment. Indeed, this body of research exposes the need to consider that each child arrives to the educational environment with a lifetime of the environment moderating that individual’s genetic predisposition. Therefore, it is unsurprising that a rank order of ability will exist, and that children will respond differently to educational interventions.

## What role can biological research play in interpreting and refining intervention research?

This paper examined how a comprehensive understanding of gene–environment interplay can help explain individual differences in outcomes of educational interventions. In what follows, we suggest how biological research can be used to reconceptualise the effects of educational interventions.

Research has shown that countries with the greatest educational equity have the highest scores on standardized measures of achievement.^110,111^ Consequently, an important goal of education systems across societies and countries is to improve equity. Improving educational equity is certainly a critical first step toward improving society. However, in addition to providing equitable opportunities for all children regardless of social status, perhaps the greatest insight we can gain from the field of biology is to embrace the existence of individual differences even when a high level of equity exists. When achieving equality in education, it is not surprising that children do not achieve at the same level, because different children require different inputs. Educational equity aims to provide individualized resources to achieve the same outcomes regardless of individual barriers and starting conditions. Therefore, the inherent goal of educational equity is for all children to perform at the same level on an educational outcome measure. However, research in the field of biology suggests that even with a perfectly equitable system, we will still find individual differences and therefore should not expect equal achievement. In other words, true equity is not attainable as we cannot expect an educational policy to bring all students to the same level. In this context, it is important to acknowledge that equity does not imply that everyone will be able to achieve the same educational outcome, it only means that each individual is provided with individualized inputs and environments.

The expectation that education can narrow the distribution of educational outcomes ignores the fact that the biological mechanisms (described above), that support learning across development, generate individual differences. Indeed, previous research that has used GWAS, GPS, gene–environment correlations, and gene–environment interaction methodologies to predict educational attainment has resulted in a large body of evidence that indicates that biological predispositions are directly linked to individual differences in educational outcomes. This means that equalizing the educational environment will not eliminate individual differences in educational achievement. Therefore, we must reconceptualise how we evaluate educational attainment. This is necessary in order to implement realistic and compassionate educational expectations and policies.

In order to re-evaluate educational attainment, it is important to first define the term educational attainment. Educational attainment is often measured as a single outcome measure, such as the average of school grades or the grade point average (GPA) that is thought to be most representative of general ability. However, there is no one distribution for “educational attainment”. Indeed, there are countless distributions (each of them closely approximating the normal distribution when considering a population of interest) for the many subtypes of skills associated with educational attainment. Several examples of possible educational measurement subtypes include math ability, reading ability, working memory capacity, or musical ability. If a student is performing very well in math, it does not automatically follow that this individual is also a strong reader or shines in art class. A long-term goal for evaluating the effectiveness of educational interventions should be to evaluate how an intervention relates to many different measures of educational achievement. Thus, instead of conceptualizing educational attainment on one distribution using a single measure such as GPA, it should be conceptualized on many different distributions. Therefore, educational attainment should be defined as an overarching term that includes many distinct measures of abilities related to education. We should expect individuals to fall along different parts of the distribution for each measure of educational attainment, depending on their strengths and weaknesses. Consequently, it is important to restructure educational policy to embrace individual differences and create a more diverse set of educational opportunities.^112^ Beyond the teaching of basic skills in reading and math that allow individuals to become successful members of society, it is important to offer students a variety of educational opportunities in order for them to be able to find niches within the educational system that best fit their genetic predispositions and experiences within which they can therefore thrive and succeed.

**Fig. 2:**
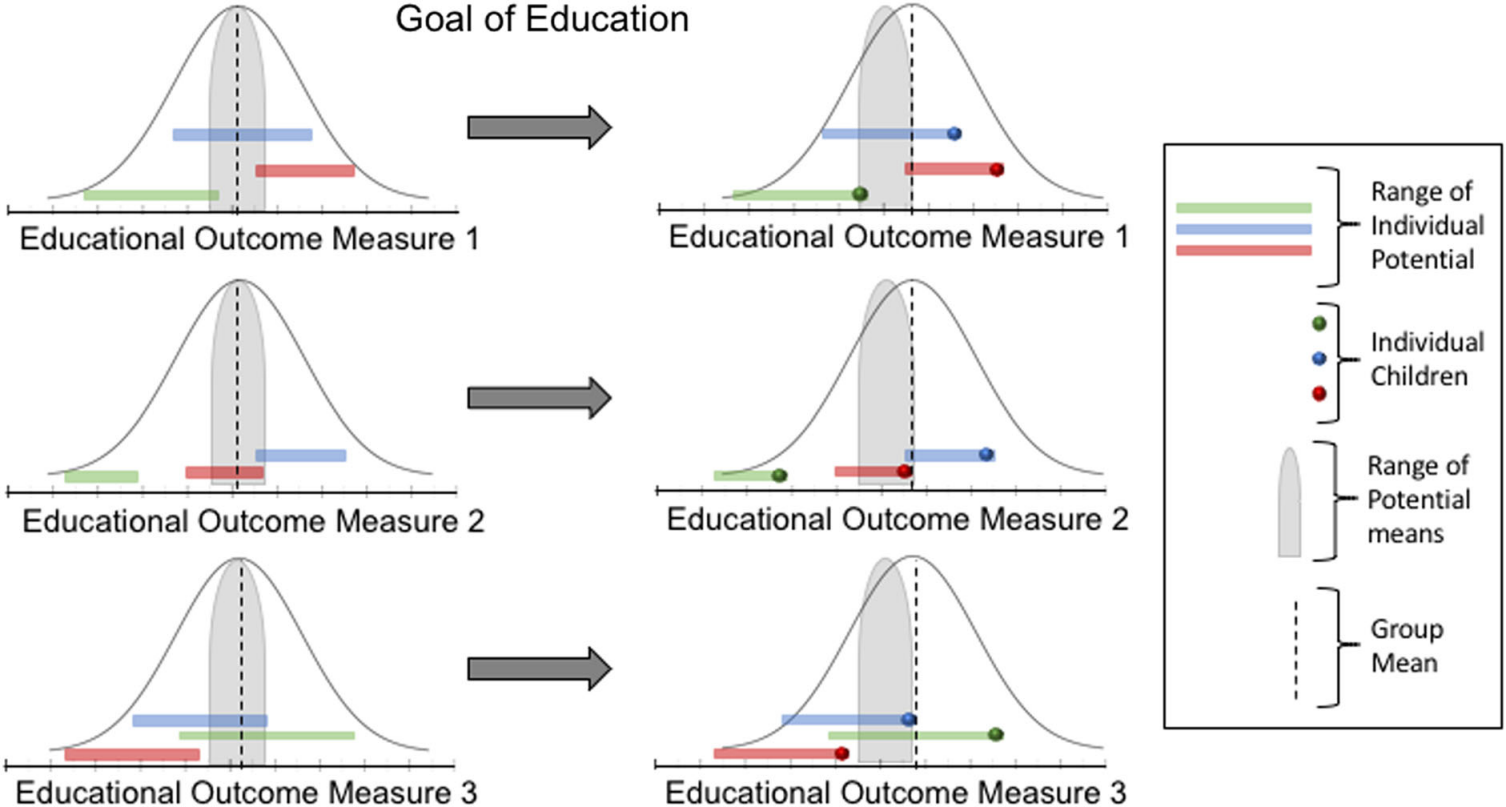
The left side of this figure depicts normal distributions of three educational outcomes measures. Individual children each have a range of scores that they may be predisposed to fall within. Ranges of three example individual children are highlighted in green, blue, and red. A hypothetical range that represents the extent to which the mean of the distribution can shift is highlighted in gray. A goal of education could be to help each child achieve the maximum score within their own individual range on these particular educational outcome measures. The right side of the figure depicts the scores of the three children and the group mean of the population if this goal of education is achieved. The educational outcome scores for the three example children are depicted with circles at the right end of each child’s range of individual potential. The dotted line on the right represents where the group mean would fall if all children achieved their maximum potential score

Finally, research on gene–environment interplay reveals that an individual’s genetic sequence should be conceptualized as a predisposition for a range of potential behavioral outcomes. In this context it has been suggested that the environment acts as a “dimmer switch” for genetic predispositions.^113^ This is important for evaluating the success of intervention research. Specifically, educational interventions should be used to help each individual child optimize their own individual range of potential (see Fig. 2). Critically, an individual’s position in a rank order will differ depending on the educational outcome measure. For example, one individual may be in the 80th percentile on numeracy but the 50th percentile in literacy. As such, there is no one rank order for an individual, but rather an infinite number of rank orders that depend on the specific educational outcome measure being considered. Having said this, we do want to acknowledge that there is a substantial and reproducible relationship between measures of IQ and educational achievement across many domains of learning. Furthermore, it is well established that IQ is heritable.^114^ However, there is research to show that IQ does not fully explain the heritability of educational outcomes.^115^ So, while it is important to recognize that IQ does predict variability in a diverse set of educational outcome measures, there do exist individual differences within and across educational domains that cannot be explained by IQ. Put differently, two individuals with the same IQ may excel in different domains of learning during their educational careers. It is these differences that we argue are necessary to consider rather than striving to equalize the performance of learners within a narrow set of learning domains. Together this suggests that an intervention that changes the mean or the position of individual children in a rank order, but does not eliminate the existence of a rank order, should not be interpreted negatively, implying that individual ability is fixed. Instead, it is time to embrace individual differences and support strengths and weaknesses through education. Additionally, education need not endeavor for every child to achieve the upper limit of their range for each educational outcome measure. Rather, beyond ensuring that children have a solid foundation in basic skills in core educational subjects, the goal of education should be to foster children’s enjoyment and motivation toward learning. In other words, a goal of educational interventions should be to maximize potential at the individual level for a wide variety of specific cognitive and emotional educational outcome measures, rather than shift the overall mean or attempt to narrow the distribution of educational achievement more broadly.

## Conclusions

The results of educational interventions intended to improve educational outcome measures are disappointing, particularly in children from impoverished environments. Researchers and policymakers have tried to improve equity in the educational environment in an effort to reduce the achievement gap between children. However, research has consistently reported that early educational interventions do not eliminate individual differences in a population.^36^ Moreover, children experience and respond to educational interventions differently. The evidence reviewed in this paper suggests that the interplay between genetic predispositions and environmental exposure across developmental time influences the way that children respond to educational interventions. For example, GWAS and GPS methodologies reveal that individual differences in genetic predispositions predict variability between children in terms of their educational achievement.^9,69,70,72^ Moreover, these stable individual differences are a consequence of biological mechanisms that support the interplay between genetic predispositions and the embedding of experience into our biology, rather than a problem with the educational environment. Currently, some conceptualize the goals of educational interventions as an attempt to both shift the mean and narrow the distribution of particular measures of educational attainment (such as GPA) that are thought to be most representative of individual ability. We recommend reconceptualising the term “educational attainment” and the goals of educational interventions. The term “educational attainment” should be an umbrella term that encompasses a wide variety of specific measurable educational outcome measures. The primary goal of educational interventions should be to maximize each child’s potential on all educational outcome measures. Notably, maximization of potential should not simply aim to optimize a child’s achievement. Instead, the role of education should be to facilitate children’s ability to select environments that align with their genotypes. Moreover, researchers, educators, and policymakers must be cognizant of the fact that each individual child enters the education system armed with a lifetime worth of interplay between their genetic predispositions and environmental exposure. Each child’s lifetime of gene–environment interplay affects the way that the child responds to education as a whole as well as to targeted educational interventions. Moreover, gene–environment interplay is a dynamic and ongoing process across an entire lifespan, not a static event such as an educational intervention. In view of this, it is senseless to assume that one type of short-term environmental exposure (such as a short-term educational intervention) will achieve long-term gains. Instead, it is probable that implementing sustained interventions will reduce fade-out effects. Therefore, we recommend that educational interventions researchers should move toward implementing changes to the educational environment that help interventions have more long-term beneficial effects (i.e., reduce fade-out effects) at the individual child level, rather than the group level. Educational interventions should identify the range of potential for each individual child and help each child achieve their potential.

Taken together, the findings reviewed above clearly suggest that, at the population level, the most important goal of educational policy is to implement equitable systems that provide individuals within the population the opportunities to reach their individual levels of achievement across a kaleidoscope of potential educational outcome. The biological research reviewed in this paper clearly demonstrates that a system that insists that all students can reach the same educational levels of achievment both within and across different educational outcomes, severely overestimates the potential of environmental effects, and therefore lacks sufficient consideration of individual differences and human diversity.
